# Apperceptive Prosopagnosia Secondary to an Ischemic Infarct of the Lingual Gyrus: A Case Report and an Update on the Neuroanatomy, Neurophysiology, and Phenomenology of Prosopagnosia

**DOI:** 10.7759/cureus.11272

**Published:** 2020-10-31

**Authors:** Hassan Kesserwani, Adam Kesserwani

**Affiliations:** 1 Neurology, Flowers Medical Group, Dothan, USA; 2 Neurology, Dothan Neurology, Dothan, USA

**Keywords:** vision disturbance, ischemic stroke

## Abstract

Phenomenology is the philosophical study of experience and its core feature of sentience, the very ability to be conscious of a sensation and how we perceive it. Nowhere is this idea more vivid, as in the phenomenon of vision and the ability to form and sense a visual percept. The clinical entity of prosopagnosia, the ability to sense but not recognize a face, strikes at the heart of this phenomenon. We describe a classic case of selective apperceptive prosopagnosia due to an ischemic infarct of the left occipital lobe with extension to the lingual gyrus. It is well-established that acquired prosopagnosia usually involves the right more than the left occipital cortex, with localization of lesions bilaterally more than unilaterally. The ischemic infarcts strategically involve the fusiform gyrus, inferior occipital gyrus, the fundus of the posterior temporal sulcus, parahippocampal gyrus, and, rarely, lingual gyrus, which is almost always not a solitary finding. We seize upon this opportunity to explore the concept of visual prosopagnosia and outline the latest ideas on the neuroanatomical localization, neurophysiology, and classification of this intriguing phenomenon.

## Introduction

Visual agnosia is a disruption of the pathway between object sensation and concept formation. By definition, there is no loss of cognition or understanding, aphasia, or a sensory deficit. This means that visual afferentation is not disrupted significantly to interfere with visual percept formation. To paraphrase, the visual percept is constructed but there is a failure of interpretation. If sentience is the ability to experience sensations and cognizance is the awareness of experiencing a sensation, then agnosia is a more difficult and nuanced concept. Historically, the subdivision of visual agnosia into apperceptive or associative varieties provided a mechanistic framework. The apperceptive variety falls into the category of sentience. The associative category falls into the category of a disconnection syndrome [1].

We propose that agnosia is best defined as a failure of integrating perception and/or association when analyzing a percept, the object of perception. This definition is all-encompassing, covering impairment of higher cortical centers, even in the absence of a conspicuous perception deficit or an obvious disconnection syndrome.

Lesions tend to involve the bilateral inferior parieto-occipital cortex. The right hemisphere is more frequently afflicted than the left hemisphere, in keeping with the lateralization of facial recognition to the right hemisphere [2]. Lesions usually involve the triad of the inferior occipital gyrus (IOG), fusiform gyrus (FG), or posterior superior temporal sulcus (pSTS). This is known as the core facial network. Recent functional magnetic resonance imaging studies (fMRI) and magneto-encephalography (MEG) studies have suggested that the processing of a visual image cascades up from the IOG to the FG [3]. Whereas the FG and IOG are more involved in facial recognition, the pSTS is more dedicated to facial expression [4].

Prosopagnosia is usually not an isolated symptom but part of a tetrad in various combinations of topographical disorientation, visual field defect, or dyschromatopsia [5]. However, it does have certain idiosyncratic features. Patients may not be able to identify familiar faces but they can match and discriminate faces. To cite an example, a patient may not be able to name or recognize a familiar face but is able to discriminate a familiar from a non-familiar face. Immediate recall and a matching task from an assortment of faces may be preserved [6].

There are some intriguing properties of face-selective cells and facial recognition. Inverting a face dramatically reduces the firing rate of face-selective cells in Macaque monkeys [7]. There are well-defined electrophysiological correlates to facial recognition and modulation. The negativity-170 (N170) event-related-potential (ERP) is both an electrophysiological surrogate marker for encoding facial recognition and processing affect [8]. The N250 ERP matches the visual percept to a specific memory [9].

The lingual gyrus is bounded anteriorly by the parahippocampal gyrus with which it is continuous, medially by the calcarine sulcus and laterally by the collateral sulcus, which separates it from the fusiform gyrus inferomedially. Superior to the lingual gyrus lies the cuneus. Prosopagnosia is almost never an isolated lesion of the lingual gyrus [6].

We present the case of a 38-year-old woman who developed a migrainous ischemic infarction of the left occipital lobe with extension into the lingual gyrus. Remarkably, she developed selective prosopagnosia of her husband's face, being able to identify him only through his voice and body morphology. Fortunately, this was a transient phenomenon, and recovery was expected as prosopagnosia is known to be more pernicious when there is bilateral involvement of the FG and/or IOG. We suspect that the transience of this phenomenon may also be due to the excellence of the collateral circulation of the occipital lobe and due to the phenomenon of diaschisis [10]. We seize upon this opportunity to explore the phenomenon of prosopagnosia and its neuroanatomy, neurophysiology, and semiology.

## Case presentation

We present the case of a 38-year-old right-handed woman who developed an acceleration of migraine with aura headaches. During a week of intense emotional trauma, she woke up one night with a horrific, non-thunderclap, stabbing headache. Her speech was slurred, and she noted loss of vision of the right side of the surroundings. A remarkable symptom was her inability to recognize faces, including her husband's who was recognized by his voice and body habitus. She was also unable to recognize her family members at her grandmother's funeral, some of which she identified by their hair. This whole episode lasted five days. She made a good recovery, except that she was left with mild residual writing mistakes, slower reading speed, and diminished visual memory. A visit to the emergency room several days later was surprisingly non-productive. A computed tomography (CT) scan showed a left occipital ischemic infarct and she was sent home on 81 milligrams (mg) aspirin daily and told to follow up with her neurologist and a cardiologist. Her neurologist obtained a thrombophilia panel, which revealed a protein S deficiency with heterozygosity of the protein S (PROS1) mutation, and decided to switch her daily aspirin to the anti-coagulant apixaban at a dose of 5 mg twice daily.

Family history included a strong history of premature coronary artery disease but no premature cerebrovascular disease or venous thrombosis. She was a non-smoker, not on estrogen-based therapy, and denied a history of deep venous thrombosis or miscarriages.

Medications included fluoxetine 40 mg daily, erenumab 140 mg subcutaneously every month, and apixaban 5 mg twice daily.

Blood pressure (BP) measured 123/86 mmHg, a pulse of 71 beats per minute, weight registered at 223 pounds, height measured 5 foot and 8 inches, and a body mass index (BMI) of 33.9. Precordial chest examination revealed no murmur and carotid auscultation revealed no bruit.

Neurological examination revealed normal gait cadence and tandem walking. Speech was fluent. Cranial nerve examination did not reveal a visual field cut or neglect with visual field confrontation. There was no evidence of visuomotor apraxia, limb-kinetic or ideomotor apraxia; being able to execute manual pantomime and transitive acts, with normal sequence motion and deftness of the fingers. The rest of the neurological examination, including power, reflexes, and sensory examination, including graphesthesia and stereognosis of the hands, was normal.

**Figure 1:**
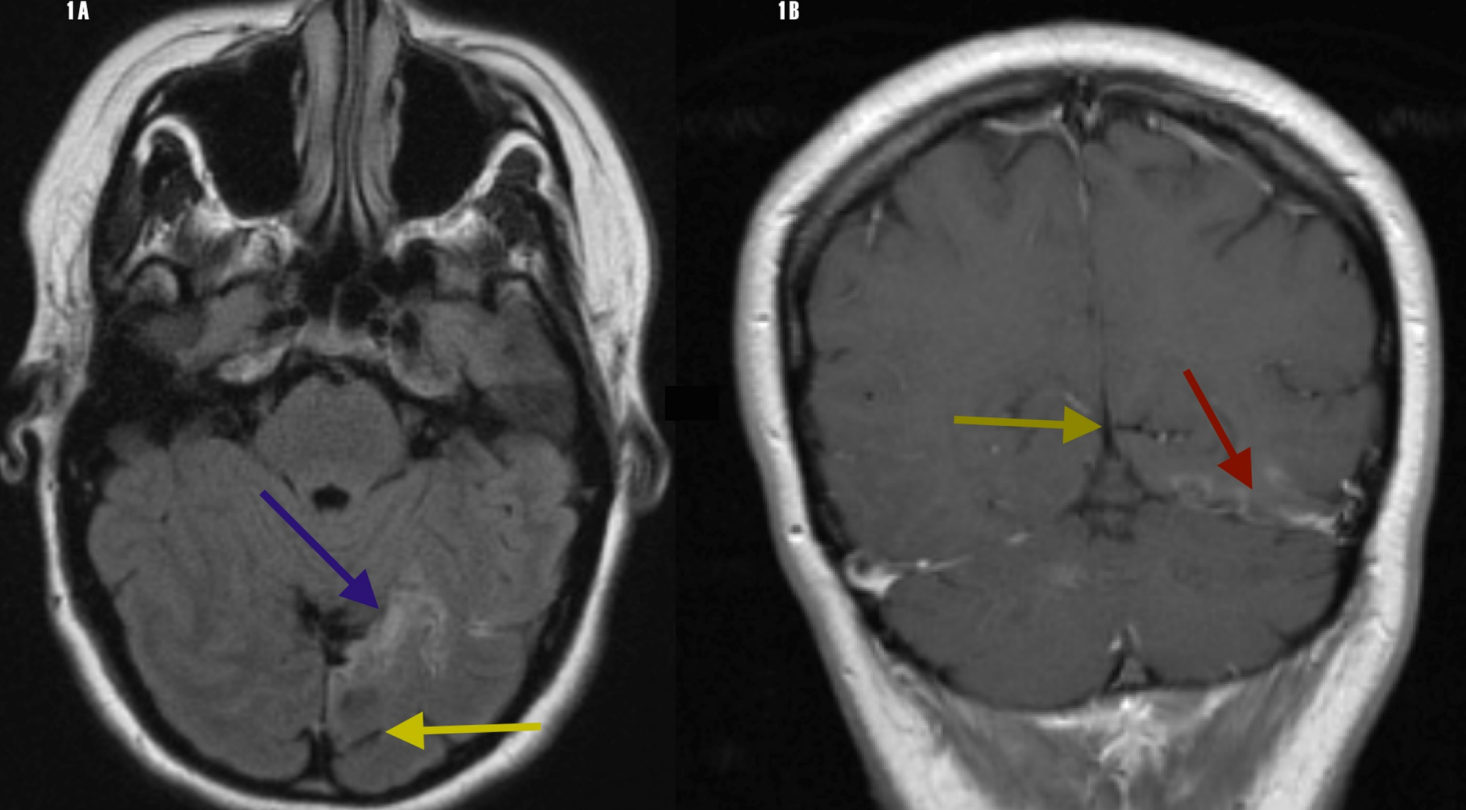
MRI: 1A. FLAIR axial image - left lingual gyrus (blue arrow) and calcarine sulcus (yellow arrow). 1B. T1 gadolinium-enhanced coronal image - left lingual gyrus (red arrow) and calcarine sulcus (yellow arrow) MRI: magnetic resonance imaging; FLAIR: fluid-attenuated inversion recovery; and T1 gadolinium-enhanced image

The patient was sent to us for further consultation 17 months after the event. We launched an embolic workup that included a transthoracic echocardiogram with intravenous air bubbles, which was negative for a patent foramen ovale. A cervical Doppler carotid duplex scan revealed no evidence of cervical carotid stenosis. A transcranial Doppler (TCD) ultrasound with insonation of the trans-orbital, temporal, and occipital windows revealed normal mean systolic velocities of the bilateral middle cerebral, anterior cerebral, posterior cerebral, internal carotid siphon, vertebral and basilar arteries, respectively. This finding was confirmed with normal magnetic resonance imaging (MRA) of the brain. A 30-day cardiac event monitor was negative for paroxysmal atrial fibrillation and a cardiac loop monitor was implanted. Fasting blood lipids were normal. A repeat magnetic resonance image (MRI) of the brain revealed an old ischemic infarct of the left lingual gyrus with extension into the left sub-cortical occipital lobe (Figure 1).

This case captured our attention because of the five-day episode of prosopagnosia. This is remarkable because she was only able to recognize her husband by his body habitus and voice and her first cousins by the patterns of their hair. We believe the extent of her ischemic infarct probably involved the striate cortex, as she also developed hemianopia with probable complete resolution. The resolution of this infarct is not unusual because of her youth, the rich collateral blood flow of the occipital lobe cortex, and probable temporary diaschisis. We believe the prosopagnosia is apperceptive because she was completely unable to both recognize faces and identify them. With associative aprosopagnosia, patients may identify a face as a face but may be unable to associate a name, an emotive experience, or a sense of familiarity. Furthermore, prosopagnosia, when it involves the lingual gyrus almost always involves the FG and IOG. This case opened up a window of opportunity for us to explore the phenomenon of prosopagnosia. The issue regarding continuing an anti-thrombotic regimen is contentious, as protein S deficiency is usually associated with venous thrombosis and not arterial thrombosis. The plan is to switch to an antiplatelet regimen if the implanted cardiac loop monitor does not reveal atrial fibrillation after six months.

## Discussion

**Figure 2:**
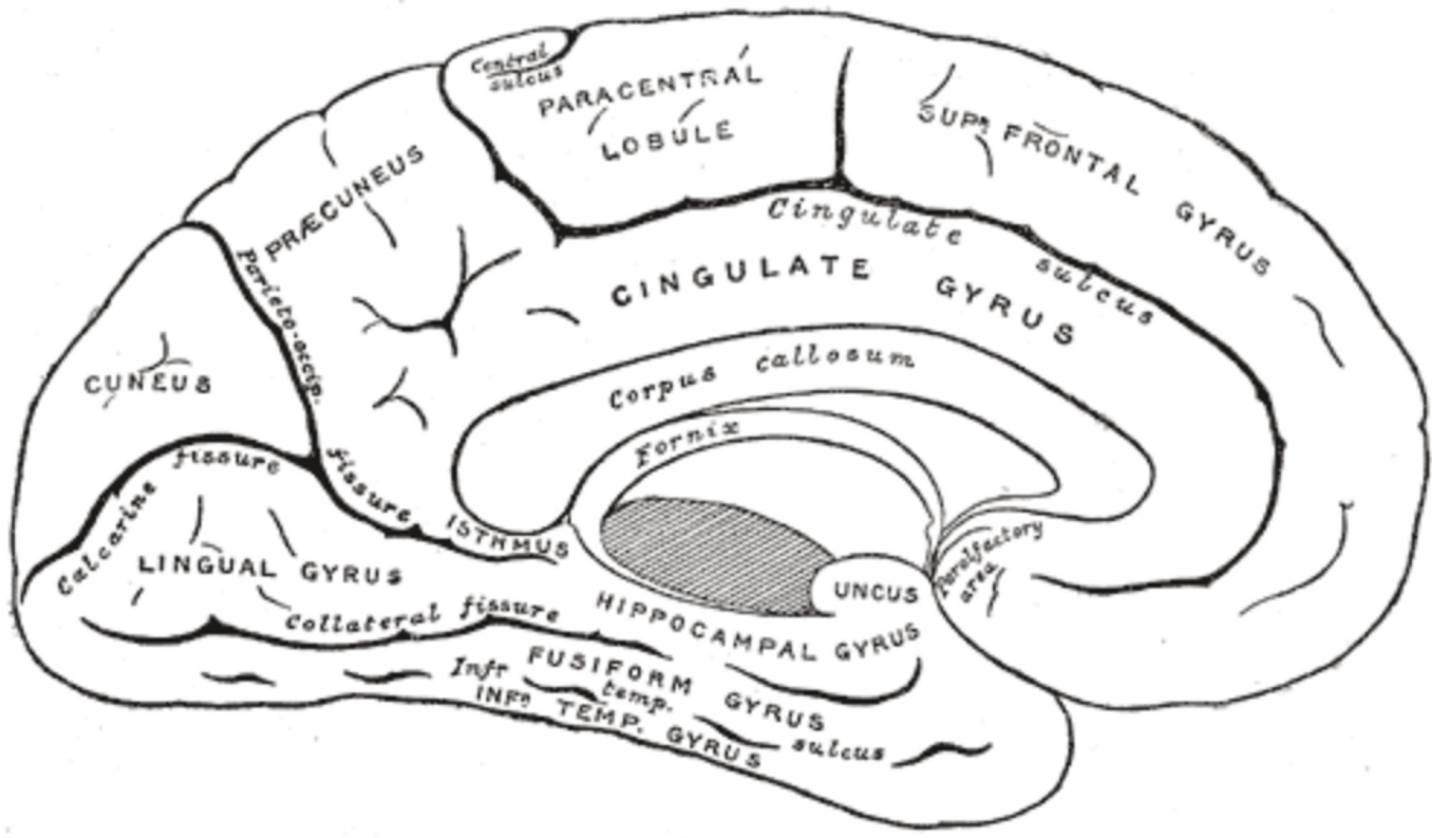
Medial sagittal view of left occipital and temporal lobes - highlighting the lingual gyrus and fusiform gyrus.

In order to orient oneself, we will first provide a sagittal view of the medial and ventral aspect of the occipital and temporal lobes in order to highlight the fusiform gyrus and lingual gyrus (Figure 2).

Prosopagnosia is divided into two major sub-groups; the acquired sub-group, which is divided into apperceptive and associative types, and developmental prosopagnosia. The neuroanatomical substrates, semiology, electrophysiology, and associated features are summarized in Table 1 [5,11]. 

**Table 1:** A clinical and neuroanatomical classification of prosopagnosia: the apperceptive and associative varieties are usually acquired Negativity (N)

APPERCEPTIVE	ASSOCIATIVE	DEVELOPMENTAL
Occipito-temporal: Mid-fusiform gyrus / Inferior occipital gyrus	Anterior temporal: Para-hippocampal gyrus	Possibly involving a connection between the occipitotemporal and anterior temporal regions: Inferior longitudinal fasciculus
Visual percept not formed despite visual input - a deficit in encoding	Visual percept formed but not matched with a feature such as emotional content or memory trace	Either apperceptive or associative deficits or both
Abnormal N170 evoked potential	Normal N170 evoked potential	Enhanced N170 evoked potential with facial inversion in the younger population
Tetrad: prosopagnosia, superior visual field defect, dyschromatopsia, and topographic disorientation	Tetrad	Genetic component

Developmental or congenital prosopagnosia is a life-long condition characterized by a poor ability to identify faces despite normal social and intellectual development. It is estimated to occur in about 2% of the population [12]. It has been an elusive condition to characterize. Facial recognition is thought to be automatic and holistic. The part-whole effect refers to the ability to recognize a face when a region of the face combined with another region leads to instant identification. Individuals with developmental prosopagnosia had problems recognizing eyes when presented with another feature but not when the mouth was presented with another part of the face. With face inversion, the younger patients showed an enhanced N170 response when compared to controls [13]. Morphometric studies have also revealed reduced right middle fusiform gyrus and inferior temporal gyrus volumes [14].

There are at least three connecting pathways in the brain; subcortical U-fibers connecting adjacent gyri, radiating fibers that fan out between lobes, and the fasiciculi or bundles of fibers. The first two are well-delineated between the temporal and occipital lobes of the brain. The third pathway, the inferior longitudinal fasciculi (IFL), was historically a contentious pathway. Diffusion tensor imaging and tractography has revolutionized this field and has confirmed the existence of the IFL, which connects the occipital and anterior temporal lobe. Disruptions of these pathways can lead to visual agnosia, such as prosopagnosia, visual amnesia to novel stimuli, and hypo-emotionality, when presented with an emotive signal, such as as a loved one's face. The trajectories and properties of the two major pathways from the visual cortex to the temporal lobe are summarized in Table 2 [15]. 

**Table 2:** Comparison of the direct and indirect occipito-anterior temporal pathways Visual (V)

DIRECT PATHWAY	INDIRECT PATHWAY
Direct projections from V4 to para-hippocampal gyrus, and from amygdala back to V2, V4	Serial, hierarchical from the extra-striate cortex to the anterior temporal lobe
Short-latency response	Long-latency response
Inferior longitudinal fasciculus	Multi-synaptic subcortical U-fibers
Carries emotional valence and visual memory	

Cortical-evoked potentials or event-related potentials are a useful electrophysiological tool for assessing cognitive function. Responses are averaged and summed after many trials; useful patterns can emerge and are useful in comparing diseased patients with controls. This technique is mostly deployed as a research tool and helps in delimiting and differentiating disease states. A brief summary of the frequently used cortical latencies and their neuroanatomical correlates are listed in Table 3 [8].

**Table 3:** Event-related potentials Negativity (N)

N170	N250
Right hemisphere	Bilateral; more robust right hemisphere
Face recognition and facial affect	Matching a face with working memory in a memory task: matching a percept to a memory
Fusiform facial area (FFA) and inferior occipital gyrus (IOG)	Posterior superior temporal sulcus (pSTS)

**Figure 3:**
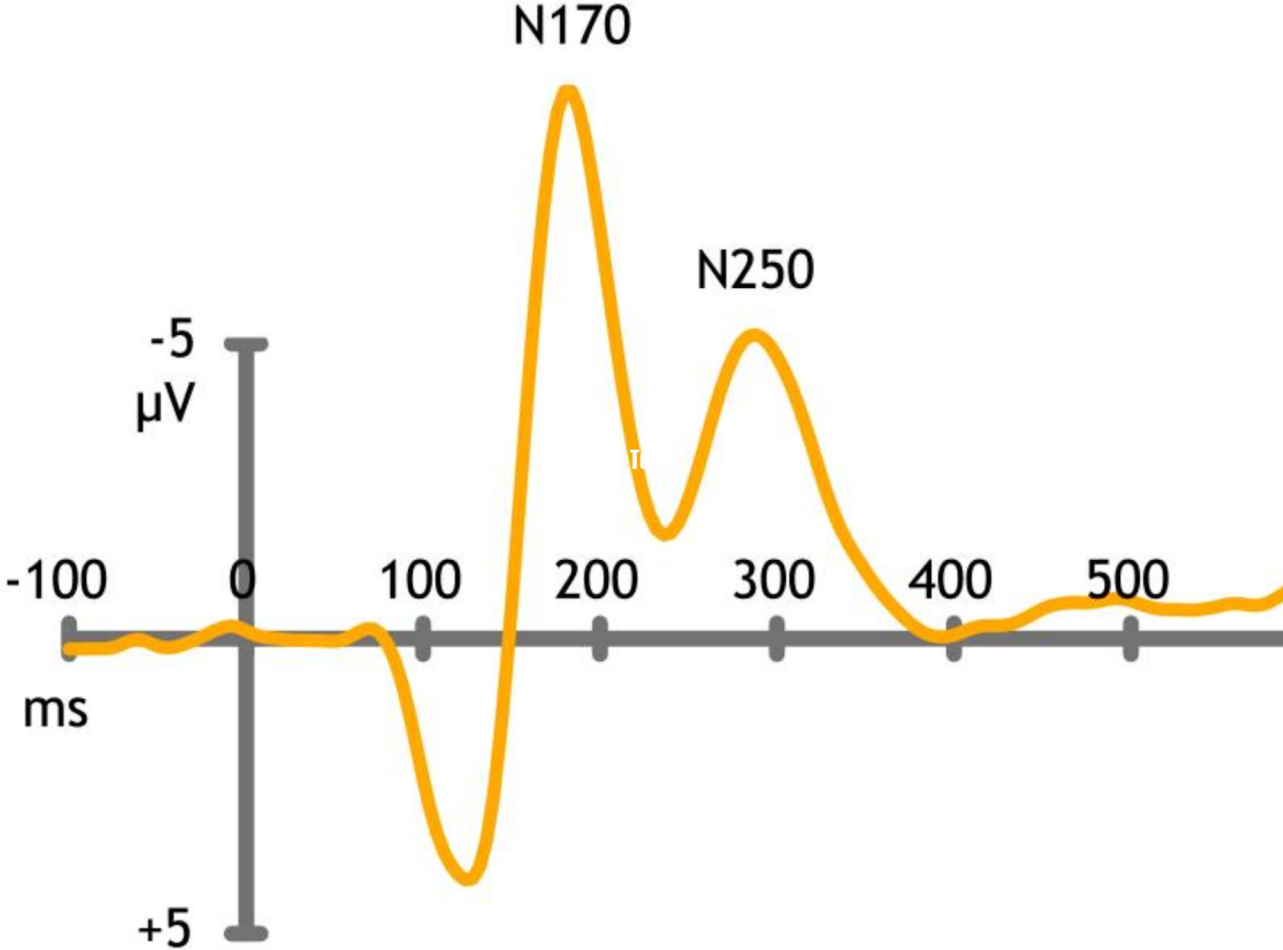
Cortical event-related potentials (ERP) N170 - facial recognition and expression. N250 - mapping face recognition to a memory trace. Negativity (N), millisecond (ms), microVolt (muV)

A typical profile when viewing a face is demonstrated below. The latency and amplitude of responses are recorded and used for comparison (Figure 3) [9].

The lingual gyrus is associated with topographic recognition, facial identification, and dreaming. Bilaterally, activation is associated with facial expressions. It is also associated with the visual recognition of words. Lesions have been associated with contralateral upper quadrantanopsias and hyperactivity with "visual snow" [16].

Semantic dementia, a subtype of frontotemporal dementia, causes progressive and substantial loss of recognition of objects. These patients are unable to connect meaning to a visual percept and appear to have fluent aphasia. One-third of the patients suffer from prosopagnosia. Imaging and pathoanatomic studies reveal bilateral anterior temporal atrophy involving the parahippocampal, peri-rhinal, fusiform gyri, and amygdala [17].

We will end with a fascinating historical note. The 1861 historical account of Phineas Gage's pre-frontal decortication and drastic change in behavior, insight, and foresight following an explosion, with an accidental rod driven through his frontal lobes, is taught early on in medical school. These anecdotal cases have provided immeasurable insights into functional neuroanatomy [18]. Less well-known is Joachim Bodamer's 1947 paper, in which he coined the term prosopagnosia. He gives us a fascinating account of three cases, one of which is a 24-year-old man, who received a bullet wound in the occipital lobes and who lost vision for a week. With gradual recovery, he developed achromatopsia and a striking loss of facial recognition. Faces appeared "tasteless" and "inert." He was unable to recognize family members, except through their voices and bodily morphological features. Remarkably, he was unable to recognize his own face in a mirror [19].

## Conclusions

We seized upon this rare case of lingual gyrus ischemic infarct to explore and update the latest findings on the phenomenon of prosopagnosia. We proposed an all-encompassing definition of prosopagnosia as an impaired integration of the visual percept and its association with a cortical feature or trace. This covers both the apperceptive, associative and developmental subtypes of prosopagnosia. In this journey, we summarized the latest findings on prosopagnosia's neuroanatomical substrates, diffuse tensor imaging, and tractography studies and streamlined the complicated event-related potential studies. We also adumbrated on its deep philosophical implications and historical importance. Understanding facial recognition has paramount importance, as it transcends disciplines and intersects with artificial intelligence and digital facial recognition.
